# Mechanisms of host adaptation by bacterial pathogens

**DOI:** 10.1093/femsre/fuae019

**Published:** 2024-07-13

**Authors:** Matthew F Barber, J Ross Fitzgerald

**Affiliations:** Institute of Ecology and Evolution, University of Oregon, Eugene, OR 97403, United States; Department of Biology, University of Oregon, Eugene, OR 97403, United States; The Roslin Institute, University of Edinburgh, Midlothian, EH25 9RG, United Kingdom

**Keywords:** bacteria, pathogen, evolution, adaptation, host, infection

## Abstract

The emergence of new infectious diseases poses a major threat to humans, animals, and broader ecosystems. Defining factors that govern the ability of pathogens to adapt to new host species is therefore a crucial research imperative. Pathogenic bacteria are of particular concern, given dwindling treatment options amid the continued expansion of antimicrobial resistance. In this review, we summarize recent advancements in the understanding of bacterial host species adaptation, with an emphasis on pathogens of humans and related mammals. We focus particularly on molecular mechanisms underlying key steps of bacterial host adaptation including colonization, nutrient acquisition, and immune evasion, as well as suggest key areas for future investigation. By developing a greater understanding of the mechanisms of host adaptation in pathogenic bacteria, we may uncover new strategies to target these microbes for the treatment and prevention of infectious diseases in humans, animals, and the broader environment.

## Teaser

This review summarizes recent studies investigating molecular and genetic mechanisms by which pathogenic bacteria adapt to infect new host species highlighting important avenues for future research.

## Introduction

**Figure 1.**
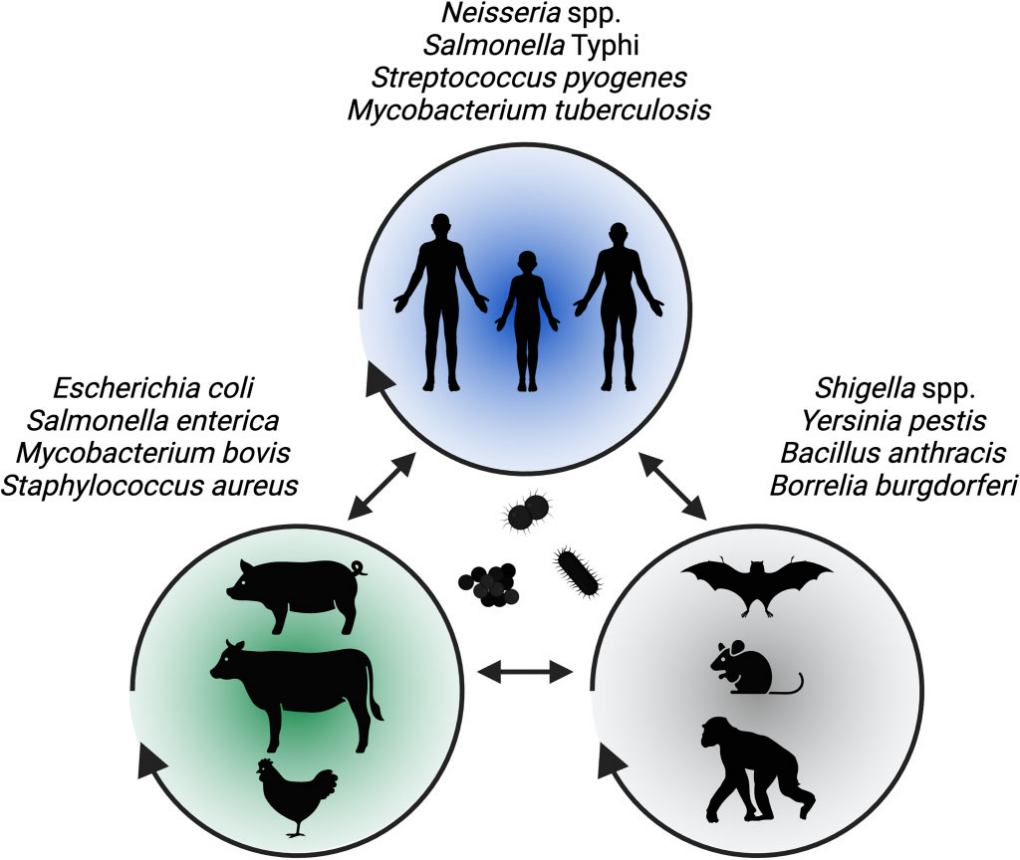
Variable host species tropism of bacterial pathogens. Examples of major bacterial pathogens restricted to humans (top), those able to transmit between humans and livestock (left), as well as zoonotic pathogens in wildlife (right). Figure created using Biorender.com.

The majority of new pathogens of humans or farmed animals originate in other animals (Woolhouse and Gaunt 2007, Karesh et al. 2012, Nelson and Vincent 2015, Haag et al. 2019). Spillover events may occur whereby pathogens are transmitted from a reservoir host into a different host species directly or via a vector, representing a zoonotic (animal to human) or anthropogenic (human to animal) infection (Fig. 1). In rare cases, spillover events may be followed by pathogen adaptation to the new host species and the capacity to transmit among members of this new host population (host switch). In order to survive and become established in a new host species, microbes must adapt to different anatomical and physiological environments with distinct immune systems and nutrient availabilities. Host genetic diversity and evolution are therefore key determinants of disease and host species tropism (host range). Zoonotic pathogens are a major global public health challenge and have led to several of the most catastrophic disease outbreaks in human history including the Black Death (caused by the bacterium *Yersinia pestis*), the 1918 influenza pandemic, the emergence of human immunodeficiency virus, and the recent SARS-CoV-2 pandemic (Gage and Kosoy 2005, Karesh et al. 2012, Lycett et al. 2019, Holmes et al. 2021). Similarly, human to animal host switches such as those by *Mycobacterium bovis* and *Staphylococcus aureus* into cattle have posed a major threat to farmed animals and wildlife populations (El-Sayed et al. 2016, Cunningham et al. 2017, Campos et al. 2022, de Souza et al. 2024). Understanding the evolutionary genetic basis and molecular mechanisms that underlie host species adaptation can identify key host–pathogen interactions underpinning disease outcomes, potentially revealing novel therapeutic targets. In addition, insights into the evolutionary events associated with host-adaptation may help us to anticipate future disease outbreaks and design effective preventive measures. While much attention has focused on host-adaptation in viruses, bacterial pathogens collectively represent a massive disease burden in humans, animals, and plants (Sundin and Wang 2018, Ramey and Ahlstrom 2020, GBD 2019 Antimicrobial Resistance Collaborators 2022). The host tropism of bacterial pathogens can also vary widely, with some restricted to a single host species and others naturally infecting a wide range of vertebrates and invertebrates (Fig. 1). Furthermore, bacterial pathogens pose a growing public health concern given the expansion and spread of antimicrobial resistance, which threatens to undermine decades of progress in global infectious disease control (Sundin and Wang 2018, Ramey and Ahlstrom 2020, Antimicrobial Resistance Collaborators 2022). Here, we review current knowledge regarding the molecular and genetic mechanisms of host-adaptation in pathogenic bacteria, drawing examples from pathogens of humans and other mammals. We focus on several key stages of pathogenesis including bacterial colonization and dissemination, nutrient acquisition, and evasion of innate and adaptive immune responses. While we briefly discuss genomic and population-level studies of host species adaptation, we direct the reader to other recent reviews in this area for a more comprehensive discussion (Sheppard et al. 2018, Matuszewska et al. 2020). We conclude by identifying open questions in bacterial pathogen host-adaptation and areas for future inquiry.

## Genetic mechanisms of bacterial adaptation

The capacity to rapidly and inexpensively sequence microbial genomes and the concurrent design of bioinformatic tools for population genomic analyses has facilitated a massive increase in understanding of how pathogens evolve over time and space (Spyrou et al. 2019, Chilambi et al. 2020, Wyres et al. 2020, Denamur et al. 2021). Phylodynamic analysis of bacterial populations has provided broad new insights into the evolutionary history of bacterial pathogens and their ability to adapt to different niches including new host species. Such studies have revealed many of the genetic processes that underpin adaptation to new host species, while subsequent functional analyses and experimental infection models can provide mechanistic insights into the impact of such events on host-adaptation.

Past studies have demonstrated that bacterial adaptation to animal hosts can occur through diverse genetic mechanisms, including single nucleotide changes, gene acquisitions and deletions, and genome rearrangements (Sheppard et al. 2018). Even single nucleotide mutations can have a profound effect on host-tropism, one example being adaptation of *S. aureus* to domesticated rabbits via a single nonsynonymous mutation in the gene *dltB* arising via a spillover event from humans (Viana et al. 2015). *dtlB* is part of an operon that decorates wall and lipoteichoic acids on the bacterial cell surface, promoting resistance to antimicrobial peptides. Identification of *dltB* mutations in other rabbit-adapted *S. aureus* strains is suggestive of convergent evolution and multiple independent spillover events from humans into rabbits (Viana et al. 2015). A separate study involving experimental adaptation of *S. aureus* to the mammary gland of sheep yielded an enrichment of nonsynonymous mutations in known virulence and colonization factors that contributed to enhanced fitness, further indicating how single nucleotide mutations can rapidly facilitate bacterial adaptation to new hosts (Bacigalupe et al. 2019). Single nucleotide changes in the *fimH* adhesin are similarly associated with host-specific serovars of the enteric pathogen *Salmonella enterica* (Yue et al. 2015). In addition, it was previously demonstrated that just two amino acid substitutions in the *Listeria monocytogenes* surface protein InlA are sufficient to enhance affinity for murine E-cadherin relative to human, a key step in bacterial host cell invasion (Wollert et al. 2007). From these and related studies it is clear that a minimal number of single nucleotide changes can be sufficient to enable major shifts in host species tropism of bacterial pathogens in the laboratory and in nature.

Horizontal gene transfer associated with both homologous or nonhomologous recombination is also a major driver of bacterial host-adaptation (Touchon et al. 2017, Arnold et al. 2022, Moura de Sousa et al. 2023). Rates and mechanisms of horizontal gene transfer vary widely across bacterial taxa and can occur via conjugation (plasmids), transduction (bacteriophages), transposons, insertion sequence (IS) elements, and phage-induced chromosomal islands (PICIs), among others (Martínez-Rubio et al. 2017, Touchon et al. 2017, Partridge et al. 2018, Rodríguez-Beltrán et al. 2021). Acquisition of a mobile genetic element itself via nonhomologous recombination can be associated with the gain of one or multiple genes that facilitate changes in host species tropism, including virulence factors (Richardson et al. 2018, Yebra et al. 2022). For example, *S. aureus* genomes carry temperate phages that encode host-specific immune modulators (Ingmer et al. 2019, Hatoum-Aslan 2021), while PICIs encode host-specific immune modulators, mediators of coagulation, and biofilm formation (Penadés and Christie 2015, Martínez-Rubio et al. 2017). Mobile genetic elements can also promote gene loss or disruption, leading to changes in pathogenicity. For example, the widespread ϕSa3int prophages in *S. aureus* encode several virulence factors of their own, but also integrate into the chromosomally encoded β-toxin gene *hlb*, leading to loss of β-toxin expression (Winkler et al. 1965, Tran et al. 2019).

Homologous recombination has also played an important role in bacterial adaptation to new host niches. Of note, a bovine subtype of *S. aureus* ST71 evolved via extensive recombination events, with older bovine lineages of *S. aureus* conferring new traits associated with immune modulation, adherence, and cellular invasion (Spoor et al. 2015). Avian strains of *S. aureus* have also evolved via recombination events that have impacted phenotypes beneficial for survival in chickens (Murray et al. 2017). A remarkable example of large-scale chromosomal events dramatically impacting host and disease tropism is provided by the *S. aureus* subspecies *anaerobius*, a unique ovine-restricted lineage causing Morel’s disease (Yebra et al. 2021). This lineage evolved from an ancestor of *S. aureus* via multiple chromosomal rearrangements, widespread IS element insertion, and extensive pseudogene formation that collectively resulted in a highly fastidious, host- and tissue-restricted subtype of *S. aureus* (Yebra et al. 2021). Loss of gene function mutations have also contributed to host-specialization among host-restricted *S. enterica* isolates, potentially relating to changes in metabolic capacity (Langridge et al. 2015). Many of the clearest examples of recombination and gene loss during host adaptation have been observed among obligate intracellular bacteria, including *Rickettsia* and other symbionts (McCutcheon and Moran 2012, Diop et al. 2018). In some cases, recombination between different symbionts as well as transfer of metabolic genes between host and bacteria genomes has led to complex and highly interdependent symbioses (Husnik et al. 2013, Husnik and McCutcheon 2016, 2018). While considerable progress has been made in understanding the genetic and evolutionary processes underpinning bacterial host-adaptation, the remaining focus of the current review will be on functional mechanisms of host-adaptation. We point the reader toward recent reviews on the genetics and genomics of bacterial host-adaptation for further discussion of these topics (Sheppard et al. 2018, Matuszewska et al. 2020).

## Colonization and dissemination

The initiation of an infection begins with colonization, and animals are colonized by diverse commensal microbes at every major epithelial barrier site including the skin, gastrointestinal tract, respiratory tract, and urogenital tract (Hooper 2015, Bäumler and Sperandio 2016, Greenbaum et al. 2019, Lee et al. 2019, Swaney and Kalan 2021, Harris-Tryon and Grice 2022). Variation in the resident microbiota between body sites reflects the unique chemical and physical environments encountered by microbes and the challenges posed by those environments. In many cases, pathogenic bacteria may also initiate colonization via a wound or other disruption of the normal epithelial barrier.

A common feature of colonization by many pathogens across body sites is attachment to host cells, extracellular matrix, or mucosa. The expression of bacterial surface molecules termed adhesins is critical for adherence to host tissues. Adhesins are typically proteins that facilitate direct interactions between the bacterial cell and host surface molecules. In many cases, genetic differences among pathogens and hosts have been shown to mediate species tropism by regulating adherence and colonization. For example, studies of *L. monocytogenes* have revealed that InlA and InlB, two surface proteins essential for host cell invasion, are both highly specific to their respective human targets. InlA binds to human and guinea pig E-cadherin protein, but does not recognize the mouse or rat orthologs (Lecuit et al. 1999). Notably, a single amino acid mutation at position 16 in E-cadherin is sufficient to determine host species tropism of this interaction (Lecuit et al. 1999). InlB conversely recognizes two host surface receptors, Met and gC1qR, both in a human-specific manner (Khelef et al. 2006). Other well-studied cases involve human-specific recognition of the polymeric immunoglobulin receptor by *Streptococcus pneumoniae* which mediates invasion of the respiratory epithelium (Zhang et al. 2000, Elm et al. 2004), as well as multiple pathogens that engage in species-specific interactions with host CD46 in the respiratory tract and skin (Okada et al. 1995, Johansson et al. 2003, 2005, Lövkvist et al. 2008, Matsui et al. 2009). Together these past studies support the notion that initial points of contact between bacterial pathogens and their hosts are crucial determinants of host species adaptation.

**Figure 2.**
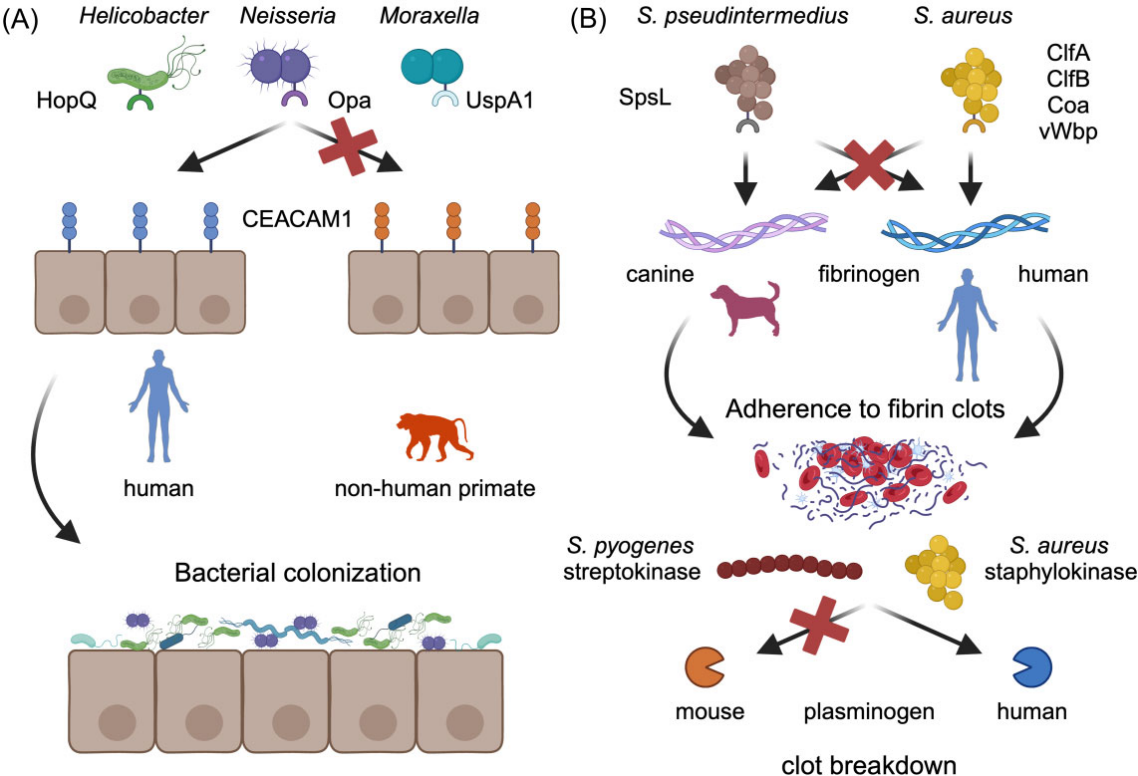
Host species adaptation impacts bacterial colonization and dissemination. (A) Human-specific *Helicobacter, Neisseria*, and *Moraxella* spp. encode surface adhesins (HopQ, Opa, and UspA1, respectively) with selectivity for human CEACAM1 relative to other nonhuman primates. Binding of bacterial adhesins to epithelial CEACAM subsequently mediates host colonization. (B) Canine and human-adapted staphylococcal species encode distinct fibrinogen-binding proteins that promote the formation and adherence to fibrin clots, contributing to abscess formation (top). Human-specific recognition of plasminogen by *S. pyogenes* streptokinase and *S. aureus* staphylokinase mediates breakdown of fibrin clots and pathogen dissemination (bottom). Figure created using Biorender.com.

Recent advances in our understanding of host-adaptation during colonization have focused on diverse bacterial adhesins that have converged on recognition of vertebrate cell carcinoembryonic antigen-associated cell adhesin molecules, or CEACAMs. CEACAM proteins are expressed on the surfaces of diverse vertebrate cell types, mediating cell–cell adhesion and signaling functions required for development and tissue remodeling (Gray-Owen and Blumberg 2006, Kuespert et al. 2006). In addition to serving these important host functions, many pathogenic bacteria have evolved CEACAM-binding adhesins as a means of colonization and invasion. Some of the first described CEACAM-binding adhesins, including Opa proteins in *Neisseria* spp. and OmpP1 in *Haemophilus influenzae*, have long been known to be selective to their respective host species (Muenzner et al. 2000, 2005, Sintsova et al. 2015, Tchoupa et al. 2015). The importance of this type of host adaptation was exemplified by studies demonstrating that CEACAM1 transgenic mice can be successfully colonized with *Neisseria meningitidis* and *Neisseria gonorrhoeae*, which are normally highly restricted to humans (Li et al. 2011, Johswich et al. 2013). Additional CEACAM-binding adhesins have recently been characterized including HopQ in *Helicobacter pylori*, the β protein in *Streptococcus agalactiae*, and the R28 protein in *Streptococcus pyogenes* (Javaheri et al. 2016, Königer et al. 2016, van Sorge et al. 2021, Catton et al. 2023). In all cases, adhesins have been found to be narrowly host-adapted (Fig. 2A). Notably, the CEACAM-binding adhesins described to date lack any structural or sequence identity with one another, indicating that recognition of host CEACAMs has emerged independently in multiple bacterial genera through convergent evolution (Sadarangani et al. 2011, Javaheri et al. 2016, Königer et al. 2016, Catton et al. 2023). The observed host-specificity of CEACAM-binding adhesins also presents a paradox, given that a single bacterial adhesin often recognizes multiple CEACAM paralogs expressed by a particular host: why is there such selectivity between CEACAM orthologs in different host species? An answer to this question has been provided by two recent studies that report evidence of repeated positive selection acting on CEACAMs in primates (Adrian et al. 2019, Baker et al. 2022). These findings are consistent with pathogen-driven evolution, in which CEACAM mutations that prevent pathogen colonization have repeatedly spread in host populations due to natural selection. In addition, we and others have observed that CEACAM paralogs are subject to frequent bouts of gene conversion, whereby extracellular domains are exchanged between gene paralogs within species (Kammerer and Zimmermann 2010, Baker et al. 2022). This combination of rapid amino acid substitutions and gene conversion produces a scenario in which multiple CEACAM paralogs within a species share higher sequence identity to one another than they do to orthologs in related species (Kammerer and Zimmermann 2010, Baker et al. 2022). In this way it is likely that pathogen-driven evolution of CEACAMs has itself contributed to entrenchment of bacterial host species adaptation.

In addition to direct interaction with host cells, pathogen colonization and dissemination can also occur through interactions with the host extracellular matrix and vascular tissues (Boyle and Finlay 2003, Vieira et al. 2014, Hammerschmidt et al. 2019). In many instances this attachment further promotes biofilm or abscess formation, exacerbating disease pathology and complicating treatment (Borlee et al. 2010, Bhattacharya and Horswill 2024). The host-specific nature of these interactions has been well-characterized in the Gram-positive staphylococci and streptococci. For example, pathogenic staphylococci can encode multiple surface proteins that bind fibrinogen, an abundant protein complex in plasma and a major component of the host coagulation cascade (Speziale et al. 2014, Foster 2019). During coagulation, thrombin cleaves the ⍺ and β chains of fibrinogen leading to the formation of fibrin clots and platelet activation (Schenone et al. 2004). Binding of bacterial surface proteins to fibrinogen allows pathogens to simultaneously interfere with coagulation, adhere to fibrin (as well as fibrinogen-binding host cells), and impair host immune responses. *Staphylococcus aureus* encodes several distinct fibrinogen-binding proteins, some of which possess restricted tropism for humans (87) (Fig. 2B). In addition, it was recently shown that the canine-adapted pathogen *Staphylococcus pseudintermedius* encodes a distinct fibrinogen-binding protein, SpsL, which is highly specific to canine fibrinogen. The recognition site for SpsL was identified as a highly polymorphic region of the fibrinogen ⍺ chain in dogs, suggesting that different breeds may exhibit differential recognition by *S. pseudintermedius* SpsL (Fig. 2B). Beyond attachment to fibrinogen, pathogens can also manipulate the coagulation cascade to their advantage. Staphylococci are classically distinguished as coagulase positive or negative, denoting the ability of some members of this genus to clot plasma (McAdow et al. 2012, Liesenborghs et al. 2018). Staphylococcal coagulase activity is mediated by at least two proteins, staphylocoagulase (Coa) and von Willebrand-binding protein (vWbp). Coagulation of plasma has been observed to be a host-specific trait (Raus and Love 1991, Friedrich et al. 2006), and recent work has demonstrated that vWbp has undergone a series of gene acquisition and loss events across the *Staphylococcus* genus phylogeny (Pickering et al. 2021). Moreover, vWbp exhibits species-specific coagulation activity, consistent with repeated host adaptation of this key virulence factor (Pickering et al. 2021) (Fig. 2B).

Paradoxically, many pathogens that promote coagulation also encode factors that break down clots, often by hijacking host plasminogen. Plasminogen is a protease present in the bloodstream that, upon conversion to its active plasmin form, cleaves fibrin and other host proteins to promote fibrinolysis and clot destruction (Keragala and Medcalf 2021, Mutch and Medcalf 2023). Plasminogen is also crucial for processes such as inflammation, leukocyte migration, and wound healing. By binding to and activating plasminogen, pathogens are able to break down clots and host extracellular matrix components to promote dissemination during systemic infection. The evolution of plasminogen-binding proteins has been observed in diverse pathogens, including *S. aureus, S. pyogenes*, and *Y. pestis* (Verhamme et al. 2015, Peetermans et al. 2016). It has been known for decades that *S. pyogenes* streptokinase is highly specific to human plasminogen (McCoy et al. 1991), hampering the application of animal infection models. The importance of this species-specific interaction was illustrated by the observation that injection of mice with human plasminogen was sufficient to enhance virulence of *S. pyogenes* (Khil et al. 2003), and that transgenic mice expressing human plasminogen quickly succumb to infection by this organism (Sun et al. 2004) (Fig. 2B).

Many factors that mediate initial host colonization of a bacterium can also promote immune evasion (Arciola et al. 2018, de Vor et al. 2020). For example, while bacterial adhesins that recognize host CEACAM proteins mediate host-specific colonization of mucosal surfaces, they also impact recognition by primate neutrophils that express “decoy” CEACAMs, which mediate bacterial opsonization and phagocytosis (Schmitter et al. 2004, Sintsova et al. 2015, Adrian et al. 2019). Balancing these selective pressures to promote colonization while simultaneously evading host immune recognition may thus contribute to the outcome of host-specific infections. Similarly, many factors that promote bacterial abscess formation simultaneously enable colonization, dissemination, as well as promoting protection against the host immune response (Cheng et al. 2011, Crosby et al. 2016, Paczosa and Mecsas 2016). Collectively these recent studies illustrate how host species adaptation can promote colonization and dissemination of diverse bacterial pathogens.

## Nutrient acquisition

Once bacteria have colonized host tissues, a key obstacle to survival is the acquisition of nutrients. While many bacteria are flexible in the range of nutrients (particularly carbon sources) they can metabolize, host species have a major influence on nutrient availability. Among the best studied group of nutrients in this regard are transition metals such as iron, zinc, and manganese, which serve as necessary cofactors for many essential bacterial proteins (Kelliher 2016, Palmer and Skaar 2016, Sheldon et al. 2016, Lopez and Skaar 2018, Gerner et al. 2020). Within the host environment, these metals are typically sequestered by metal-binding proteins as part of physiological metal homeostasis. For example, transferrin family proteins are secreted in nearly all extracellular fluids in animals, where they bind tightly to free iron to prevent redox chemical reactions and tissue damage (Anderson and Frazer 2017, Hsu et al. 2022). The sequestration of nutrient metals by host proteins therefore presents a major barrier to bacterial growth. The importance of metals for bacterial pathogenesis has also been highlighted by studies demonstrating the requirement of bacterial metal acquisition systems *in vivo* (Skaar et al. 2004, Garcia et al. 2011, Deriu et al. 2013, Juttukonda et al. 2017, Spiga et al. 2023). Furthermore, hosts can actively enhance metal sequestration by upregulating metal-binding proteins during infection or at barrier tissues, leading to the concept of “nutritional immunity” as an important host defense strategy against cellular pathogens (Weinberg 1975, Schryvers 1999, Skaar and Raffatellu 2015, Cornelissen 2018, Murdoch and Skaar 2022).

**Figure 3.**
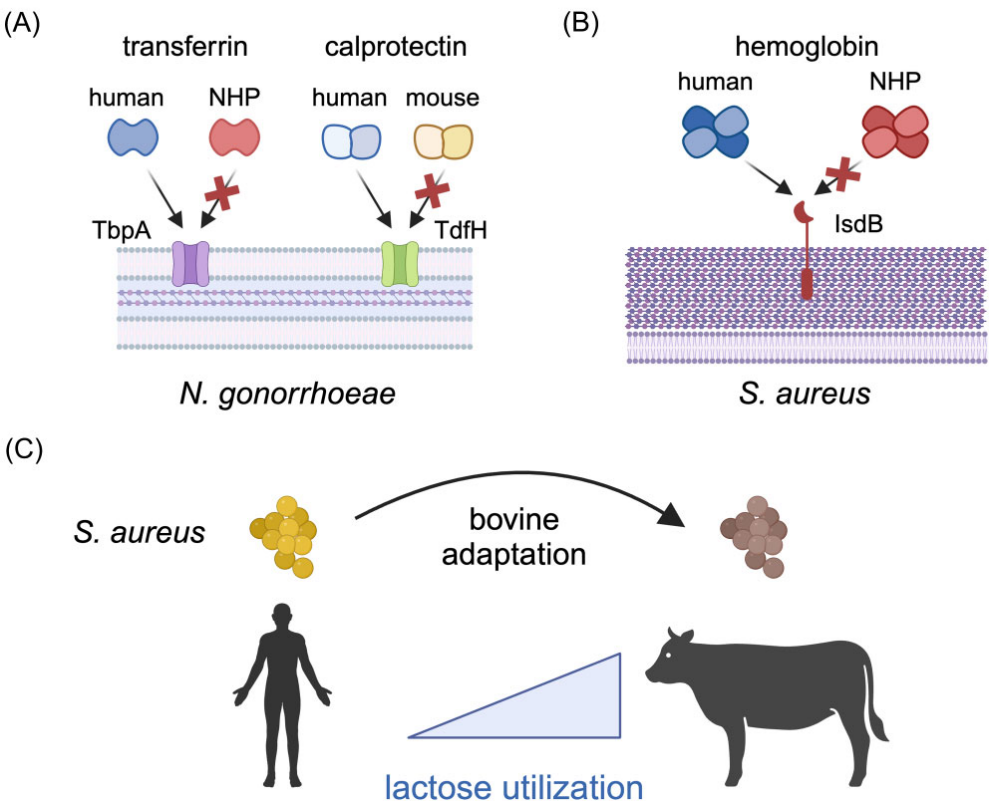
Evolution of host-specific nutrient acquisition by bacterial pathogens. (A) Surface receptors (TbpA and TdfH) in *N. gonorrhoeae* exhibit narrow host specificity for metal-binding proteins (transferrin and calprotectin) from humans relative to nonhuman primates (NHP) or mice. (B) The hemoglobin receptor IsdB from *S. aureus* selectively scavenges heme from human hemoglobin relative to NHPs and rodents. (C) Adaptation of *S. aureus* lineages to dairy cattle has led to enhanced utilization of lactose, an abundant nutrient source during bovine mastitis. Figure created using Biorender.com.

Bacterial metal acquisition systems are diverse and frequently involve the secretion of small molecules termed siderophores that can effectively compete with host proteins for metals (Holden and Bachman 2015, Kramer et al. 2020, Antelo et al. 2021). Metal-bound siderophores are subsequently reacquired by bacteria via surface receptors to mediate nutrient acquisition. In addition to siderophores, other metal-acquisition strategies involving bacterial cell surface receptors that directly bind to host metal-binding proteins may be employed (Palmer and Skaar 2016, Cornelissen 2018, Ostan et al. 2021, Murdoch and Skaar 2022). These bacterial receptors are often selective for a narrow range of host species (Schryvers and Morris 1988, Schryvers and Gonzalez 1989, Cornelissen et al. 1992, Schryvers and Gray-Owen 1992), and recent work has revealed how mechanisms of metal acquisition may contribute to the restricted species tropism of bacterial pathogens. For example, we previously demonstrated that both transferrin and lactoferrin exhibit signatures of repeated natural selection in humans and nonhuman primates (Barber and Elde 2014, Barber et al. 2016). In the case of transferrin, rapidly evolving regions of the protein match closely with the binding surface of transferrin-binding protein A (TbpA), a TonB-dependent receptor encoded by several pathogenic Gram-negative species including *N. gonorrhoeae, N. meningitidis*, and *H. influenzae* (Noinaj et al. 2012, Morgenthau et al. 2013, Pogoutse and Moraes 2017). These findings suggest that bacterial iron acquisition has driven host adaptation, in turn restricting the tropism of bacteria that rely on these host iron-binding proteins (Barber and Elde 2015, Pogoutse and Moraes 2020) (Fig. 3A). Subsequent studies have similarly identified evidence of host adaptation in other bacterial metal acquisition systems, including those targeting hemoglobin. Roughly 70% of the iron in the human body is bound within red blood cells in the hemoglobin protein complex as the porphyrin cofactor heme (Choby and Skaar 2016). This makes hemoglobin an abundant target for pathogen iron-acquisition systems, particularly microbes that enter the bloodstream. For example, the hemoglobin receptor IsdB in *S. aureus* had previously been shown to bind human hemoglobin more effectively than mouse (Pishchany et al. 2010). More recently, molecular studies demonstrated that just a few highly divergent substitutions in the hemoglobin ⍺ and β subunits are sufficient to enhance specificity of IsdB for hemoglobin from different nonhuman primates (Choby et al. 2018) (Fig. 3B). Adaptation to hemoglobin as a nutrient source has even been observed for *Pseudomonas aeruginosa*, a major human pathogen that is generally viewed as emerging from environmental reservoirs. During long-term chronic infections, however, *P. aeruginosa* can undergo multiple genetic alterations that enhance survival in the human host (Winstanley et al. 2016, Planet 2022), including increased iron acquisition from hemoglobin during infection of cystic fibrosis patients (Marvig et al. 2014). Collectively these recent findings highlight the role for iron acquisition in host adaptation by diverse pathogenic bacteria (Fig. 3A and B).

In addition to iron, recent work has revealed evidence of host-specific adaptation for acquisition of other key nutrient metals. Calprotectin is a protein heterodimer composed of S100A8 and S100A9 subunits and is among the most abundant proteins in neutrophils, released in high concentrations at sites of infection (Corbin et al. 2008, Kehl-Fie and Skaar 2010). Calprotectin also contributes to host nutritional immunity through its ability to sequester both zinc and manganese (Kehl-Fie and Skaar 2010, Zygiel and Nolan 2018). Evidence that bacteria can scavenge metals from calprotectin itself has previously been scarce, but recent work revealed that *N. gonorrhoeae* encodes an outer membrane receptor, TdfH, which binds calprotectin to mediate zinc scavenging (Jean et al. 2016, Kammerman et al. 2020, Bera et al. 2022). Notably, TdfH is selective toward human calprotectin in comparison to that of other mammals, indicating that this human-specific pathogen may have evolved to specialize for zinc acquisition from its native host (Fig. 3A).

Carbohydrates, like metals, are crucial for microbial survival within animal hosts. While many bacteria possess the ability to uptake and metabolize a variety of carbon sources, the availability of these nutrients can vary substantially both within host tissues as well as between species (Richardson 2019, Lee et al. 2022, Pokorzynski and Groisman 2023, Potter and Criss 2023). As previously highlighted, *S. aureus* provides an informative model for studying pathogen host-switch events given that it has had a long-term association with humans while also undergoing repeated transfers to other animal hosts. In particular, *S. aureus* is a major cause of bovine mastitis in dairy cattle and imposes a significant economic and veterinary healthcare burden (Haag et al. 2019, Campos et al. 2022). While transmission of *S. aureus* between humans and cattle is well-documented, recent work has begun to uncover the mechanisms that mediate successful host-switching by this pathogen. A study by ourselves and others revealed that genes under positive selection in host-adapted lineages of *S. aureus* are enriched for numerous metabolic genes, including metal and carbohydrate transporters (Richardson et al. 2018). It was also observed that bovine mastitis isolates exhibit enhanced utilization of lactose, a major source of carbohydrates in the bovine mammary gland (Richardson et al. 2018) (Fig. 3C). These results indicate that bovine-associated *S. aureus* lineages have undergone genetic attenuations to enhance nutrient acquisition in their transfer from humans to cattle. The importance of carbohydrate metabolism during *S. aureus* infection was similarly demonstrated by researchers who recently found that elevated glucose levels in murine diabetes models simultaneously dampened the host neutrophil response to infection while also enhancing *S. aureus* virulence within the host (Thurlow et al. 2020). Although it has been known for decades that diabetes patients are at increased risk for a variety of infections (Chávez-Reyes et al. 2021, van Niekerk et al. 2021), this work has aided in clarifying the impacts of glucose metabolism on both host and pathogen. While it is clear that carbohydrate availability within hosts plays crucial roles in colonization and infection, how host-specific metabolites impact species adaptation remains an important area for future investigation.

Examples of host adaptation for nutrients beyond metals and carbohydrates in bacterial pathogens likely remain to be discovered. For example, a previous genome-wide association study in the multihost gastric pathogen *Campylobacter* implicated vitamin B5 biosynthesis as a key factor in adaptation to bovine hosts (Sheppard et al. 2013). Bovine-adapted strains exhibited enhanced growth *in vitro* when vitamin B5 was limited, supporting this hypothesis. Many studies to date have demonstrated that metabolism and nutrient acquisition are key features during other ecological transitions, such as invasion of the bloodstream or urinary tract as well as successful competition within the animal gut (Bäumler and Sperandio 2016, Passalacqua et al. 2016). It is, therefore likely that we have only scratched the surface in our understanding of the connection between nutrient acquisition and host-adaptation in bacterial pathogens.

## Cell-autonomous immune evasion

The capacity for bacterial pathogens to avoid clearance by the host immune system is critical for the outcome of infection. Characterized mechanisms of immune evasion in pathogenic bacteria are plentiful, reflecting their key importance in disease pathogenesis and intense research in this area (Okumura and Nizet 2014, Koymans et al. 2017, Quillin and Seifert 2018, Bengoechea and Sa Pessoa 2019, de Jong et al. 2019, Chandra et al. 2022). Likewise, instances of host species adaptation in immune evasion pathways are among the best characterized in this field relative to other interactions. Given the bounty of excellent work in this area, we will focus our discussion on a subset of recent studies as well as a small number of classic works to place these findings in context.

**Figure 4.**
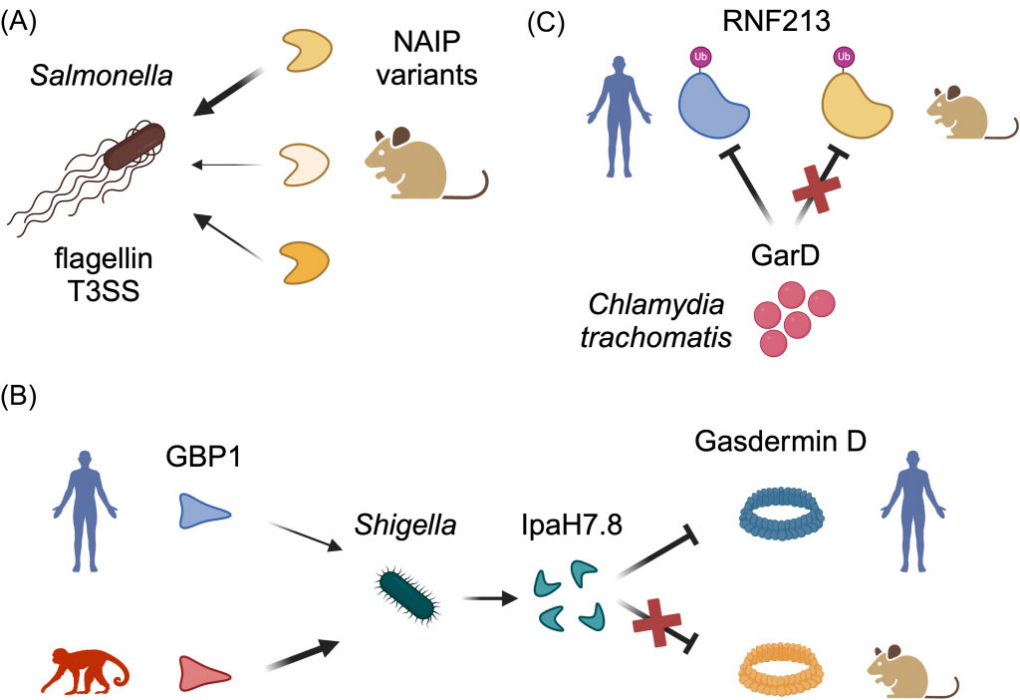
Host species tropism dictates bacterial cell-autonomous immune evasion. (A) Rodents encode a wide range of distinct NAIP genetic variants with differing abilities to recognize bacterial ligands. NAIP-inflammasome activation is differentially activated by *Salmonella* ligands, including both flagellin and T3SS components. (B) The cytoplasmic bacterial pathogen *S. flexneri* is recognized by interferon-stimulated GTPases including GBP1. Variants of GBP1 in New World primates exhibit enhanced detection of *Shigella* relative to humans (left). In addition, the *Shigella*-secreted effector IpaH7.8 is able to inhibit formation of gasdermin D pore during induction of host cell pyroptosis in a host-species specific manner (right). (C) The GarD protein from *Chlamydia trachomatis* is able to block immune recognition by human cells, but not those of rodents. Detection of *C. trachomatis* in humans was recently shown to be mediated by the E3 ubiqutin ligase, RNF213. Figure created using Biorender.com.

Under the broad umbrella of immune evasion, recent years have seen notable progress in our understanding of cell-autonomous immunity. These innate immune responses are particularly relevant to defense against intracellular bacteria, viruses, and parasites, and are employed by diverse host cell types. Key host factors involved in cell autonomous immunity are inflammasomes, multiprotein complexes that assemble in response to various intracellular cues (Broz and Dixit 2016, Rathinam and Fitzgerald 2016, Brewer et al. 2019, Mitchell et al. 2019). These cues can include the cleavage of proinflammatory caspases triggered by upstream immune signaling, direct detection of microbe-associated molecular patterns (MAMPs) such as lipopolysaccharide (LPS) (von Moltke et al. 2013), as well as the activities of pathogen effectors such as proteases (von Moltke et al. 2013, Shin and Brodsky 2015). Inflammasomes comprise a shared structure, which includes either nucleotide-binding oligomerization domain-like receptor (NLR) or AIM2-like receptor (ALR) proteins with inflammatory caspases, a class of proteases that mediate diverse processes including inflammation and programmed cell death (Man et al. 2017, Rathinam et al. 2019). Inflammasome activation leads to assembly of the complex as well as cleavage and activation of associated caspases, triggering release of inflammatory cytokines and a form of cell death termed pyroptosis. Genetic and molecular studies over the last decade have greatly improved our understanding of both inflammasome function and the role of inflammasomes in infectious and inflammatory diseases (von Moltke et al. 2013, Shin and Brodsky 2015, Broz and Dixit 2016, Rathinam and Fitzgerald 2016, Mitchell et al. 2019). One of the best studied examples involves inflammasome activation by bacterial flagellin and type-3 secretion system (T3SS) proteins. Infection by diverse bacterial pathogens, including *Salmonella* and *Legionella* spp., leads to activation of the NLRC4 inflammasome due in part to cytoplasmic flagellin detection by NLR apoptosis inhibitory protein 5 (NAIP5) (Franchi et al. 2006, Miao et al. 2006, Molofsky et al. 2006, Lightfield et al. 2008). Notably, a previous study leveraged a panel of chimeric mouse NAIP proteins to pinpoint the mechanisms defining ligand binding (Tenthorey et al. 2014) (Fig. 4A). Rodent genomes encode a large number of related NAIP genes that exhibit unique microbial ligand specificities for bacterial flagellin and T3SS proteins, likely reflecting long-term counter-adaptation by both bacteria and their mammalian hosts (Kofoed and Vance 2011, Zhao et al. 2011, Tenthorey et al. 2014, Zhao et al. 2016). Given that ligand binding is required for NAIP inflammasome assembly, it is likely that the specific combination of NAIP variants in a host would have important consequences for host species tropism. Indeed, more recent work has demonstrated that NAIP-NLRC4 inflammasome activation in gut epithelial cells is sufficient to protect mice from infection by *Shigella flexneri*, an important gastrointestinal pathogen of humans (Suzuki et al. 2014, Mitchell et al. 2020). Inactivation of the inflammasome in turn leaves mice susceptible to infection by *Shigella*, uncovering important insights on barriers to host species adaptation and providing an important new animal model for human disease (Mitchell et al. 2020). Recent work has also demonstrated that human and murine NAIP-NLRC4 inflammasomes differentially sense and defend against intracellular *Salmonella* infection through detection of the T3SS proteins, again highlighting species-specific consequences of host variation in inflammasome components (Naseer et al. 2022, Egan et al. 2023) (Fig. 4A).

In addition to host-adaptation related to inflammasome activation, a number of recent studies have demonstrated how other aspects of cell-autonomous immunity contribute to bacterial pathogen host tropism. One key group of host factors in this regard are the guanylate-binding proteins (GBPs), which encompass a family of vertebrate cytoplasmic GTPases first identified due to their massive upregulation in response to interferon signaling (Randow et al. 2013, Tretina et al. 2019, Kutsch and Coers 2021). Vertebrates typically encode a range of distinct GBP paralogs, with humans possessing seven. Molecular studies have shown that subsets of GBPs contribute to cell-autonomous immunity by directly recognizing intracellular pathogens within pathogen-containing compartments, leading to a range of outcomes including impairment of pathogen replication, inflammasome activation, and pyroptosis (Pilla-Moffett et al. 2016, Kutsch and Coers 2021). How particular GBP paralogs contribute to defense against distinct pathogens has been an ongoing research focus. A series of studies recently demonstrated that mammalian GBP1 contributes to defense against cytoplasmic Gram-negative bacteria by directly recognizing bacterial LPS (Kutsch et al. 2020, Santos et al. 2020). We and others also found that genetic variation in a C-terminal domain of GBP1 and GBP2 termed the polybasic motif (PBM) modulates pathogen recognition in a species-specific manner (Kohler et al. 2020). Notably, the GBP1 PBM from several nonhuman primates demonstrated enhanced binding to *S. flexneri* relative to human or other mammalian GBP1 orthologs (Fig. 4B). Complementary studies have observed different requirements for human and mouse GBP1 in defense against *Legionella pneumophila*, where GBP1 can contribute to both recognition of *Legionella* containing vacuoles as well as direct lysis of the bacterium itself (Bass et al. 2023). How GBP genetic variation contributes to differences in disease outcomes between host species remains an important outstanding area for investigation.

Host innate immunity can also target bacteria that reside within intracellular compartments, a common stealth strategy employed by pathogens including *Salmonella, Legionella, Chlamydia*, and *Mycobacteria* spp. (Omotade and Roy 2019). Recent studies have revealed mechanisms by which recognition of pathogen-containing compartments contribute to host defense and influence host tropism in pathogenic bacteria. For example, the murine-adapted pathogen *Chlamydia muridarum* is cleared from human cells primed with interferon, but human-adapted *Chlamydia trachomatis* is resistant (Haldar et al. 2016). This species-specific difference in immune evasion recently enabled researchers to develop a genetic screen identifying GarD as a novel *C. trachomatis* protein that mediates resistance to cell-autonomous defenses (Fig. 4C). GarD functions by blocking ubiquitin decoration of bacteria-containing endomembrane compartments (inclusions) that would normally lead to bacterial clearance (Walsh et al. 2022). Moreover, analysis of a human cell line library identified polymorphisms in the ubiquitin ligase RNF213 that regulate defense against *C. trachomatis*, leading to the discovery that GarD defends inclusions from recognition by RNF213 itself (Walsh et al. 2022) (Fig. 4C). These findings elegantly demonstrate new mechanisms employed by pathogens to evade host cell-autonomous defenses as well as how host and pathogen genetic variation can mediate host-species adaptation.

Bacterial pathogens have also been shown to interfere with the terminal stages of pyroptosis in a host-specific manner. Gasdermins are a widely conserved family of pore-forming proteins that are activated in response to inflammasome activity and other inflammatory signals (Kovacs and Miao 2017, Broz et al. 2020). Recent work has shown that *S. flexneri* is able to inhibit pyroptosis in infected cells through the activity of IpaH7.8, a ubiquitin ligase that targets gasdermin D for degradation by the proteosome (Luchetti et al. 2021). Gasdermin D degradation is also species-specific, effective against humans but not mice (Fig. 4B). Leveraging the previously described NLRC4-deficient mouse model, researchers further demonstrated that deletion of gasdermin D in this background further enhances susceptibility to infection by human-adapted *Shigella* (Luchetti et al. 2021). Together these studies provide a snapshot of the recent progress that has been made in understanding how bacterial pathogen immune evasion mechanisms contribute to host-species adaptation.

## Humoral immune evasion

Bacteria that enter the bloodstream or other extracellular host environments must contend with a wide range of soluble, or humoral, immune defense mechanisms including antimicrobial peptides, enzymes, toxic fatty acids, circulating antibodies, and components of the host complement system (Lambris et al. 2008, Gallo and Hooper 2012, Perez-Lopez et al. 2016, Sebina and Pepper 2018). Furthermore, recognition by one or more of these humoral immune defenses often leads to recruitment of host immune cells (leukocytes), further contributing to microbial clearance. Evidence for the importance of these circulating immunity factors comes from multiple sources, including genetic susceptibility of individuals harboring mutations in humoral defense genes as well as decades of molecular studies (Lewis and Ram 2020, Conti et al. 2022).

Evidence for the role of humoral immune evasion in bacterial host species adaptation is plentiful for the complement system. Complement encompasses a large network of soluble and cell-surface proteins (over 30 in humans) that coordinate to recognize foreign bodies and recruit additional immune effectors and cells (Lambris et al. 2008, Hajishengallis et al. 2017). For many pathogens, particularly Gram-negative bacteria, soluble complement proteins are sufficient to induce bacterial lysis through assembly of a protein pore termed the membrane attack complex into the bacterial outer membrane. Complement components are abundant in serum but also present in many extracellular fluids such as tears, mucosal secretions, and saliva (Ricklin et al. 2010, Sahu et al. 2022). The complement system also interfaces with multiple other aspects of host immunity, for example by generating MAMPs detectable by other host receptors, triggering cytokine production, as well as being activated by the presence of pathogen-specific antibodies (Ricklin et al. 2010). Given this wide range of effects, it is not surprising that successful pathogens have evolved multiple mechanisms to evade or neutralize the complement system. However, the coevolution of hosts in response to pathogens has likely contributed to genetic incompatibilities that limit the effectiveness, and therefore species tropism, of complement evasion mechanisms.

**Figure 5.**
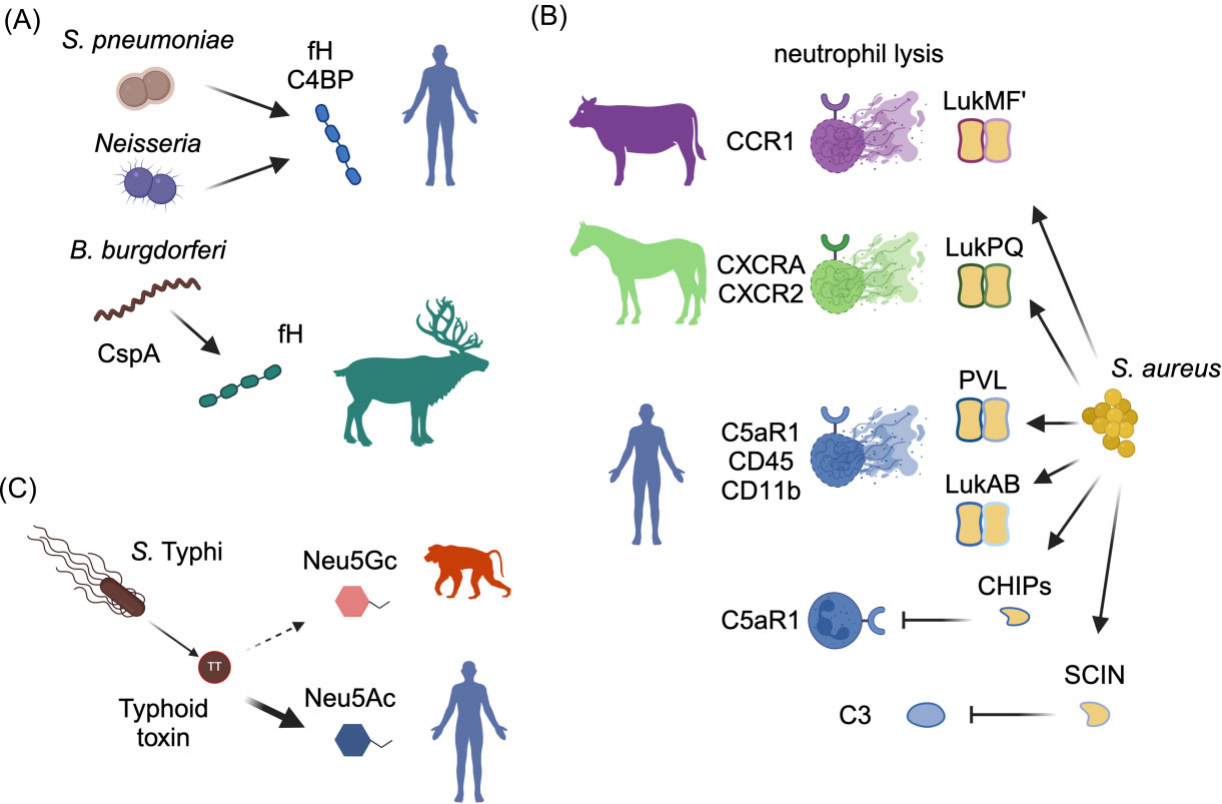
Host species adaptation of bacterial toxins and humoral immune evasion factors. (A) Surface proteins in diverse bacterial pathogens bind to host complement regulators, including fH and C4BP, to mediate evasion of complement proteins. Specificity of surface proteins for particular host complement regulators has been described in many bacteria. (B) Diverse secreted toxins and immune evasion proteins encoded by host-adapted lineages of *S. aureus*. (C) The typhoid toxin of *Salmonella* Typhi selectively binds the host surface glycan Neu5Ac, which is abundant on human cells, over Neu5Gc, which is present on most other animal species. Figure created using Biorender.com.

One of the most common strategies of microbial complement evasion involves recruitment of regulatory proteins that normal serve to protect host cells and tissues from inappropriate complement activation. Two such proteins, factor H (fH) and C4 binding protein (C4BP), are abundant in animal serum and provide protection against the alternative and classical complement pathways respectively. Pathogen surface proteins that bind fH and C4BP have been reported in diverse bacteria, including *Neisseria* spp., *Streptococcus* spp., *H. influenzae*, and *Borrelia burgdorferi* (Lambris et al. 2008). Complement regulatory proteins are highly genetically diverse between vertebrate species, limiting the species tropism of these strategies. For example, human-adapted *Neisseria* spp. can inhibit complement activation by human serum but not that of other mammals (Schneider et al. 2007, Lewis and Ram 2020) (Fig. 5A). Host adaptation related to complement evasion was elegantly illustrated by a recent study leveraging distinct species of pathogenic *Borrelia*, the causative agents of tick-borne Lyme disease (Hart et al. 2021). The authors found that the distinct host tropism of different *Borrelia* species was determined by host-specific recognition of fH by the bacterial surface protein CspA. Variation in both fH and CspA between hosts and pathogen strains directly correlated with complement evasion and the ability to transmit to distinct mammalian or avian hosts via a tick blood meal. Phylogenetic analysis of CspA further suggests that binding to host fH has evolved repeatedly across *Borrelia* via convergent evolution, illustrating the importance of complement evasion in facilitating host adaptation of this pathogen (Hart et al. 2021) (Fig. 5A).

In addition to disguising themselves through recruitment of host complement regulators, pathogens also encode factors that disrupt the complement system to promote immune evasion. For example, *S. aureus* produces a number of secreted proteins that function to bind and antagonize complement components, several of which are illustrated in Fig. 5(B). These include the prophage-encoded chemotaxis inhibitory protein of *S. aureus* (CHIPS), which binds to host C5a receptor and formylated peptide receptors to disrupt chemotaxis (de Haas et al. 2004), as well as the staphylococcal complement inhibitor (SCIN), which binds to the complement C3 convertase complex to prevent cleavage and activation (Rooijakkers et al. 2005). *Staphylococcus aureus* CHIPS and SCIN have long been known to exhibit specificity for their human targets limiting the application of murine models for studies of their role in pathogenesis (Fig. 5B). A more recent study was the first to identify a SCIN homolog in an equine-adapted *S. aureus* lineage (de Jong et al. 2018). This SCIN variant is located on a prophage exclusively associated with equine-associated *S. aureus* strain, and exhibits a broad host range against equine, human, and pig C3 convertase (de Jong et al. 2018).

Another key component of humoral immunity faced by bacterial pathogens is the presence of neutralizing antibodies. Binding of antibodies to bacterial surfaces leads to a range of downstream immune functions including complement activation, aggregation, and enhancing pathogen uptake by phagocytes (opsonization). Bacteria can counter host antibodies via a range of mechanisms, including production of proteases that cleave host antibodies. In particular, it has been known for nearly a half century that several pathogenic bacteria produce proteases that cleave human IgA, the major form of antibodies present at mucosal barriers. IgA proteases are present in both Gram-negative and Gram-positive pathogens, including *H. influenzae, N. gonorrhoeae, N. meningitidis*, and *S. pneumoniae* (Mehta et al. 1973, Plaut et al. 1975, Mulks et al. 1980). In all of these cases, bacterial IgA proteases are highly specific to humans, which contributes in part to the difficulty of generating informative animal models for these pathogens. Another example is provided by staphylococcal protein A (SpA) produced by *S. aureus* that nonspecifically binds to mammalian IgG via the Fc region immunoglobulin-binding domains in the SpA protein, thereby promoting resistance to phagocytosis (Falugi et al. 2013, Bear et al. 2023). However, chickens, for which *S. aureus* is also an important pathogen, produce a distinct structural variant of IgG, known as IgY, which is not recognized by SpA. Therefore, SpA does not have a known role in humoral immune evasion during the pathogenesis of chicken infections. Of note, it has been demonstrated that a common clone of *S. aureus* associated with global infections of broiler poultry has lost the capacity to produce SpA via a nonsense mutation in the *spA* gene (Lowder et al. 2009). Collectively these studies illustrate how evasion of humoral immunity factors contribute to host species specificity in diverse bacterial pathogens.

## Bacterial toxins and superantigens

Pathogenic bacteria are often distinguished from more benign relatives by the capacity to produce potent toxins with the ability to damage host cells or tissues (Otto 2014, Barnett et al. 2015, Moayeri et al. 2015, Chandrasekaran and Lacy 2017, Spaan et al. 2017). While many toxins are classically studied for their ability to kill leukocytes and promote immune evasion, toxins can also facilitate nutrient acquisition by lysing red blood cells in the bloodstream (Spaan et al. 2015) or mediating metal acquisition in the gut (Rivera-Chávez and Mekalanos 2019). Toxins are also crucial for the transmission of many enteric and respiratory pathogens (Viswanathan et al. 2009, Laabei et al. 2015, Rudkin et al. 2017). As such, the host species tropism of toxins are often inexorably linked to pathogen fitness. While many toxins have been shown to possess a restricted tropism for particular hosts, a number of recent studies have uncovered new mechanisms and downstream consequences of toxin host-adaptation.

Staphylococci are again notable for their production of a wide range of toxins including the family of leukocidins (Fig. 5B). Leukocidins are bi-component pore-forming toxins that are capable of lysing host leukocytes and other cell types (Seilie and Bubeck Wardenburg 2017, Spaan et al. 2017, Tromp and van Strijp 2020). In recent years, it has been shown that many staphylococcal pore-forming toxins target host cells through recognition of specific host cell surface receptors (Inoshima et al. 2011, Alonzo et al. 2013, DuMont et al. 2013, Spaan et al. 2015, Lubkin et al. 2019). Several leucocidins are encoded by prophages, and selectivity of a toxin for the receptor of its native host can promote host-adaptation via phage acquisition (Koymans et al. 2017). A number of studies have also illustrated the importance of host tropism for distinct leukocidins, such as LukMF’, which binds specifically to the chemokine receptor CCR1 expressed on bovine neutrophils (Vrieling et al. 2015) and LukPQ elaborated by *S. aureus* from horses that binds to CXCRA and CXCR2 receptors on equine neutrophils (Koop et al. 2017) (Fig. 5B). While the *S. aureus* Panton–Valentine leucocidin (PVL) and HlgCB have both previously been shown to specifically target human C5aR1 (Spaan et al. 2013, 2014), a more recent study demonstrated that mice expressing C5aR1 are susceptible to HlgCB but not PVL (Tromp et al. 2018). This surprising observation led to the discovery that the F component of PVL interacts with host CD45, which is required for full activity of the toxin and contributes to host specificity (Tromp et al. 2018). Another notable recent study focused on LukAB, a *S. aureus* leucocidin that targets host cells through recognition of the surface protein CD11b. LukAB is able to effectively bind and target human CD11b-expressing cells, but not murine cells. Using a combination of biochemical assays and phylogenetic analyses, the authors identified a critical region of CD11b responsible for LukAB binding and host species tropism. Moreover, generation of a “humanized” mouse containing the human version of this CD11b domain was sufficient to promote susceptibility of mice to *S. aureus* bloodstream infections, consistent with the activity of LukAB (Boguslawski et al. 2020) (Fig. 5B). This study provides an elegant example of how investigating host species tropism can yield important information regarding host–pathogen interactions, as well as guide the development of new animal infection models. Staphylococcal toxins have, as demonstrated by the studies highlighted here, provided a wealth of insights regarding mechanisms of host adaptation mediated by receptor-binding tropism.

An additional example of toxin host species adaptation is provided by the *Salmonella* Typhi typhoid toxin. Unlike other strains of *S. enterica*, which typically have a broad host range and are major causes of gastroenteritis, *S*. Typhi is an exclusively human pathogen that causes life-threatening typhoid fever (Dougan and Baker 2014, Galán 2016). Entry of the typhoid toxin protein complex into host cells is dependent on recognition of host cell surface sialic acids. Notably, humans lack the enzyme CMP-*N*-acetylneuraminic acid hydroxylase (CMAH), which is produced in other vertebrates and catalyzes the conversion of *N*-acetylneuraminic acid (Neu5Ac) to *N*-glycolylneuraminic acid (Neu5Gc) (Varki 2009). As a result, human cells are highly enriched for Neu5Ac glycans compared to other vertebrates. Previous work has shown that *S*. Typhi typhoid toxin exhibits strong selectivity for Neu5Ac relative to Neu5Gc, consistent with human-specific adaptation (Deng et al. 2014). Supporting this hypothesis, mice constitutively expressing CMAH exhibit enhanced resistance to typhoid toxin (Deng et al. 2014). More recent work has extended these findings to investigate the mechanism of host cell recognition by ArtAB, a related toxin produced by *Salmonella* Typhimurium, which possesses a broad mammalian host range. Biochemical and structural studies demonstrated that the ArtAB toxin is capable of recognizing both Neu5Ac and Neu5Gc, consistent with the promiscuous ecology of *S*. Typhimurium (Gao et al. 2017). In addition to toxin tropism, it is also worth noting here that host glycan interactions are important for other aspects of bacterial colonization and pathogenesis. For example, human-adapted *Neisseria* strains specifically engage with human sialic acid receptors (siglecs) via surface glycans and porin proteins, which contribute to host immune suppression (Landig et al. 2019).

Investigations of host-adaptation in bacterial toxins have also recently illustrated how pathogens leverage mimicry to manipulate host cell functions. For example, enteric pathogens including enterotoxigenic *E. coli*, as well as *Vibrio* and *Yersinia* species, encode a related family of heat stable enterotoxins. These toxins are short peptides that mimic host guanylin hormones, which are major regulators of intestinal fluid homeostasis in animals (Wang et al. 2019). Binding of guanylins or enterotoxin STa to guanylate cyclase (GC-C) on enterocytes in the gastrointestinal tract, triggering cyclic GMP production and ultimately leading to the secretion of water into the intestinal lumen. High levels of STa expressed by enteric pathogens leads to a massive activation of GC-C resulting in diarrheal disease in infected patients (Wang et al. 2019). A recent study found that STa peptides exhibit variable host species tropism, consistent with rapid divergence of the GC-C extracellular domain across primates and bats (Carey et al. 2021). The authors further identified evidence that guanylins and their cognate GC-C receptors appear to be coevolving across species, suggesting that repeated host adaptation of bacterial toxins drives the ongoing diversification of this conserved signaling pathway.

Superantigens (SAgs) are a distinct class of microbial toxins that function by activating host T cells via the T cell receptor (TCR) cross-linked to the major histocompatibility complex (MHC) proteins on antigen presenting cells (Tam and Torres 2019, Hurst et al. 2021). Binding to both receptors leads to T cell activation even in the absence of antigen, which leads to a massive proinflammatory cascade, systemic cytokine release, and inflammatory pathology. This nonspecific activation of the host adaptive immune response can promote immune evasion by overriding any pathogen-specific responses, as well as contributing to transmission or dissemination, particularly in the case of enterotoxins (Pinchuk et al. 2010). Like other toxins, SAgs have been demonstrated to exhibit selectivity for their native host species with differing affinities for T cell subsets defined by the variable region (Vβ) of the T cell receptor (Deringer et al. 1997). In the case of *S. aureus*, individual strains can express up to a dozen unique SAgs some of which have broad activity for T cells from different host species, in contrast to others that are more host-restricted in their functionality (Wilson et al. 2018). Recently we and others systematically characterized the distribution, activity, and host specificity of SAgs expressed by bovine-adapted *S. aureus* (Wilson et al. 2018). The presence of Sag genes on host-specific mobile genetic elements further supports the notion that certain SAgs have evolved for preference of particular host species. These findings thus demonstrate how host adaptation of bacterial toxins and SAgs contributes to species-specific patterns of infectious disease susceptibility among mammals.

## Concluding remarks

The broad impact of ongoing human activities including industrialization, agriculture, and modern medicine on pathogen emergence suggest that bacterial infections, including zoonoses, will continue to be a major global health concern in the 21st century. In particular, human land use and climate change are predicted to promote zoonoses and host-switch events and exacerbate infectious disease burdens in humans, plants, and animals, threatening food security and biodiversity (Gebreyes et al. 2014, Cavicchioli et al. 2019). Understanding the factors that mediate host species adaptation in pathogenic bacteria is crucial for combatting these challenges. While this review covers an array of mechanisms underpinning host species adaptation, our understanding of the basis for this process in different pathogens is limited. For example, while transmission is a crucial part of the lifecycle in all pathogens, the evolution of bacterial transmission mechanisms and their role in host adaptation is comparatively less understood (Robinson et al. 2018, 2021). It is important to emphasize that identifying adaptations required for successful transmission and establishment in a new host species will also reveal critical host–pathogen interactions that represent novel therapeutic targets in multiple-host species. For example, the identification of mutations in *dltB* that facilitated the host-switch of *S. aureus* from humans into rabbits, in parallel saw the identification of *dltB* as a novel antimicrobial target for treating infections (Pasquina et al. 2016). Similarly, extensive research into the critical role of leukocidins in the host-specific pathogenesis of human and animal diseases has led to novel therapeutics that target leukocidins to treat bacterial infections (Tam et al. 2020, Fernandez et al. 2022).

The last decade has also greatly expanded our understanding for the role of resident commensal microbes (the microbiome) in susceptibility to infectious disease (Buffie and Pamer 2013, Parlet et al. 2019, Rogers et al. 2021). The importance of the microbiome in facilitating or impairing host species adaptation in pathogens remains an important area for continued work. Investigations into the competitive interactions between different bacterial species within the human and animal microbiome are revealing many new natural antimicrobial agents, some of which may be applied for the control of infectious diseases. In particular, the increasing impact of the global AMR crisis means that additions to our armoury of antimicrobials for treating infection are urgently needed.

While this review has focused on genetic and molecular determinants of bacterial adaptation to distinct host species, genetic variation within host and pathogen populations is also a key factor in infectious disease dynamics and has been reviewed more extensively elsewhere (Smith and Sassetti 2018, Gibbs et al. 2022, Dekker 2024, Randolph et al. 2024). It is notable that many genes known to impact host species tropism are also variable within host populations, including CEACAMs, Toll like receptors, MHC loci, and others (Mikacenic et al. 2013, Parnas et al. 2015, Pdelorenze et al. 2016, Adrian et al. 2019, Baker et al. 2022, Smith et al. 2022). Population genetic studies have been particularly informative in dissecting human-specific adaptation of pathogens such as *Mycobacterium tuberculosis*, the leading cause of human infectious disease related deaths worldwide (Mortimer and Pepperell 2014, Smith et al. 2016, 2022, Gagneux 2018, Orgeur et al. 2024). Population-based approaches will also aid in the study of host-restricted pathogens for which experimental models are limited, such as *Treponema pallidum*, the causative agent of human syphilis (Houston et al. 2015, Šmajs et al. 2018, Djokic et al. 2019, Lithgow et al. 2020). Future work that continues to bridge our knowledge of within and between host adaptation will provide an improved understanding of infectious disease susceptibility across biological scales. In addition, while our review has focused primarily on pathogens of humans and related mammals, emerging studies from other animal and plant systems will aid in providing a comprehensive view of bacterial host adaptation across diverse taxa (Wiesmann et al. 2023). Collectively the studies discussed here reflect major advancements in our understanding of host species adaptation by bacterial pathogens, yet it is clear that many fundamental questions remain to be addressed in the years ahead.
